# Meeting the Sexual and Reproductive Health Needs of Internally Displaced Persons in Ethiopia’s Somali Region: A Qualitative Process Evaluation

**DOI:** 10.9745/GHSP-D-21-00818

**Published:** 2022-10-31

**Authors:** Kathryn A. O’Connell, Tesfaye Shiferaw Hailegebriel, Danielle Garfinkel, Jenna Durham, Bereket Yakob, Jemal Kassaw, Addisalem Titiyos Kebede

**Affiliations:** a EngenderHealth, Washington, DC, USA.; b EngenderHealth, Addis Ababa, Ethiopia.; c College of Health Sciences and Medicine, Wolaita Sodo University, Sodo, Ethiopia.

## Abstract

**Introduction:**

Meeting the sexual and reproductive health and rights (SRHR) needs of internally displaced persons (IDPs) is critical. Despite increased prioritization and coverage of sexual and reproductive health (SRH) services in humanitarian settings in recent decades, significant unmet needs remain. In Ethiopia, there are more than 2 million IDPs, an estimated 40% of whom have unmet need for modern contraceptives. To address this, EngenderHealth implemented a model of SRHR programming in Ethiopia’s Somali region. We share the lessons learned from this project to improve access to SRH services among IDPs.

**Methods:**

In 2021, an independent research team implemented a qualitative process evaluation among 13 key informant interviews (KIIs) with health system actors, local government partners, and organizations, and 4 focus group discussions (FGDs) with community members and community health volunteers. The team selected participants purposively following the maximum variation sampling technique and analyzed the data in NVivo 12. The team used KII and FGD guides to explore and understand what was implemented, which stakeholders were engaged in the processes and how, what was achieved, and the barriers and facilitators in implementation.

**Results:**

Contributions to project achievements included strong partnerships and stakeholder engagement, an enabling environment for SRHR, improving health worker capacity, and flexibility and adaptability. Challenges included a fragile security situation, retention of providers, and difficulty in accessing gender-based violence services, exacerbated by the coronavirus disease (COVID-19) pandemic.

**Conclusion:**

Our article offers guidance for organizations and government entities seeking to design and implement SRHR programs in humanitarian settings. Findings highlight the importance of prioritizing SRHR programming in IDP settings and illustrate adaptable activities to assist with project implementation and minimize operational challenges.

## Abstract (precis)

We share lessons learned from a project to improve access to sexual and reproductive health services among internally displaced persons in Somali region, Ethiopia.

## Teaser

**Key Findings:**

- Maintaining strong partnerships, cocreating activities with local stakeholders, coordinating with the existing health system, and aligning interventions with national priorities are key to effectively addressing the sexual and reproductive health (SRH) services needs within communities of internally displaced persons (IDPs).
- Fragile security conditions, retention of trained providers, and barriers toward sexual and gender-based violence services had detrimental roles in improving access to SRH services among IDPs, which were exacerbated by coronavirus disease (COVID-19).
- Lessons and successful approaches identified by those closest to the project, including IDPs who received services, health facility staff, and local government partners and partner organizations working in this space, offer a path for effective prioritization of sexual and reproductive health and rights (SRHR) within IDP and similar humanitarian contexts.

**Key Implication:**

- Program implementers should prioritize SRHR in IDP settings. The results of this process evaluation add to the evidence base on the effective delivery and implementation of SRHR programming for IDPs in humanitarian crises.

## INTRODUCTION

Internal displacement is a challenging, urgent, and growing humanitarian crisis. In 2020, conflict and disasters triggered 40.5 million new internal displacements globally.^1^ Internally displaced persons (IDPs) are those who have been forced to leave their homes but have remained within the borders of their country of origin. Host country governments often struggle to adequately provide basic services such as health care to these IDPs due to the competing priorities of the conflict or disaster that has displaced their citizens. Further, as IDPs lack the status of refugees and the associated protections, many multilateral agencies and nongovernmental organizations cannot offer the same resources that they provide to refugee populations.

Ethiopia has one of the highest numbers of IDPs in the world, largely due to conflict, political instability, and scarce environmental resources as a result of drought and flooding.^2^ As of July 2021, there were an estimated 3.95 million people displaced across the country with 1.8 million living in camps or camp-like settings.^2^ Women, girls, and adolescents are disproportionately impacted by internal displacement and face a high risk of physical and sexual violence, exploitation, and abuse.^3^ The crises have created urgent needs, including sexual and reproductive health (SRH) information and services, for IDPs and their host communities. Although Ethiopia as a nation has made notable strides in SRH over the past decades, these crises threaten the progress that has been made. Furthermore, Ethiopia has systems and infrastructure in place that differ from neighboring countries, notably countries that are more routinely plagued by humanitarian crises, making the nation less prepared to address essential SRH service provision among IDPs.

The Somali region is the second largest in the Federal Republic of Ethiopia, comprising Ethiopia’s third largest ethnic group. The semiarid region has a long history of clan, resource-based, and regional conflict, remaining relatively underdeveloped when compared to the other regions of Ethiopia. Culturally and economically, the region also retains a unique way of life, with seminomadic pastoralist communities and clan links playing the most important social and political unit of social organization. The local population is primarily dependent on pastoralism, and livestock is the main pillar of livelihood, supporting about 86% of the population.^4^ Evidence indicates that the Somali region has one of the worst health-seeking behavior and service utilization indicators nationally. The sexual and reproductive health and rights (SRHR) needs in the region are critical: in 2019, the Somali region had the lowest modern contraceptive use (3.4%) among married women, the second highest teenage pregnancy (22%) next to Afar, the lowest antenatal care coverage (30.2%), the lowest institutional delivery rate (23.0%), and the lowest postnatal care utilization in the first 2 days of delivery (10.3%) in the country.^5^ Furthermore, a rapid assessment implemented in 2019 among internally displaced women aged 15–49 years living in the Somali region of Ethiopia provided important insights on the demographics and the SRHR needs of this IDP population.^6^ The study found extremely high rates of illiteracy (96.7%) among respondents, and 60.7% had given birth to 5 or more children. Almost all respondents (87.7%) reported ever hearing about SRH services, but only 36% knew where to access SRH services and 91.3% did not currently use any contraceptive method. These findings are concerning given that lack of access to and utilization of contraceptive services can result in increased maternal and infant morbidities and mortalities, as well as social and psychological trauma risks within IDP communities.^7^ Addressing the SRHR needs of IDPs is critical to protecting human rights, mitigating infectious diseases, and addressing gender inequalities and violence that persist within IDP camps.^8^

Evidence indicates that the Somali region has one of the worst health-seeking behavior and service utilization indicators nationally.

Furthermore, persistent conflict, violence, and climate-induced IDP displacement have triggered widespread insecurity and movement of populations who are often forced to abandon their farms and livelihoods. Many IDPs stay with host families or spontaneously formed settlements with few, if any, basic services. In Ethiopia, IDPs have been one of the population groups most vulnerable to coronavirus disease (COVID-19) infections because of poor access to handwashing facilities, sanitation and hygiene, and limited tools to protect themselves from COVID-19.^9^ The situation is further compounded by several social and cultural barriers. Recent evidence from the Somali region illustrates that men are the leaders of the family, make most health-related decisions, and often do not support family planning. In addition, the use of contraception is seen as taboo in the region. Other obstacles to using contraception among women include fear that their husband can marry other women if they adopt a method, fear of side effects from contraception, and fear of stigma from the community.^10^

Though the prioritization and coverage of SRH services in humanitarian settings have expanded over the last few decades, significant needs remain unmet.^11^ Humanitarian organizations providing essential emergency support to IDPs often lack the necessary staff, specialized experience, and resources to address the SRHR needs of IDPs.^12^ Additionally, development organizations that wish to work with IDPs may lack the experience and expertise to navigate SRHR programming in crisis settings. These challenges can result in large gaps in ensuring the quality of urgent and lifesaving services for IDPs, especially women, girls, and adolescents. To meet the complex needs of IDPs, organizations serving these populations must coordinate and integrate traditional humanitarian services (basic services, including shelter, food, and protection) and traditional development services (welfare, education, health, social, and economic).^13^^,^^14^ This approach has been referred to as the humanitarian-development nexus, and currently, there is momentum to better understand the ways that this framework can be implemented most efficiently.^7^^,^^8^^,^^15^^–^^17^

To meet the complex needs of IDPs, organizations must coordinate and integrate traditional humanitarian and development services.

To respond to the critical SRHR needs of IDPs in the Somali region of Ethiopia, EngenderHealth implemented the Sexual and Reproductive Health and Rights for Internally Displaced Persons (SRHR-IDP) project, which aimed to ensure that IDPs, particularly adolescent girls and women in areas of highest need, have improved access to comprehensive and gender-equitable SRHR information and services. The project also sought to establish partnerships with existing government structures and partners working in humanitarian settings in Ethiopia to inform, mobilize, and influence local stakeholders to prioritize SRHR in crisis situations.

In 2021, EngenderHealth implemented an independent qualitative process evaluation to share the lessons learned from the project to improve access to SRH services among IDPs. The objective of this research was to document, explore, and share the processes involved in designing and implementing the project, as well as the lessons learned. We undertook this research to develop a case study to investigate how organizations can adapt their development approaches to humanitarian settings in response to the SRHR needs of IDPs. We also assessed the progress, contributions, and challenges of the project. Furthermore, we aimed to identify recommendations to address IDPs’ SRHR needs regarding interventions and advocacy at the regional and national levels. The results of this process evaluation add to the evidence base regarding the effective delivery and implementation of SRHR programming for IDPs and other populations in humanitarian crises. This study can also offer lessons for other development organizations working in similar humanitarian settings, especially with IDP populations.

## PROJECT DESCRIPTION

Since July 2019, EngenderHealth has been implementing the SRHR-IDP project, in the Somali region of Ethiopia within existing structures, including national and regional government and partner systems, to create urgency around the SRH needs of IDPs, particularly girls and women. Building on EngenderHealth’s experience in the implementation of SRHR programming and support for service delivery, the project adapted EngenderHealth’s activities to enable IDPs to access a continuum of care for comprehensive SRHR. This care included contraception, maternal care, and prevention and clinical management of gender-based violence (GBV). The project has 3 main objectives: (1) to ensure that IDP communities, particularly adolescent girls and women in areas of highest need, have improved access to comprehensive and gender-equitable SRHR information and services; (2) to enable existing government structures and partners working in humanitarian settings in Ethiopia to inform, mobilize, and influence local stakeholders to prioritize SRHR in crisis situations; and (3) to generate evidence to address unanswered questions pertaining to the SRHR needs and responses in humanitarian settings, particularly for IDPs. The project ended in December 2021.

The SRHR-IDP project designed activities using the socioecological model^18^ to create change at the individual, interpersonal, community, institutional, and policy levels. Project activities included establishing new national and regional partnerships; supporting community mobilization to increase awareness of and demand for SRH services, including GBV; enhancing referral pathways; improving the availability of equipment and logistics for SRH services at temporary IDP clinics; and enhancing health care providers’ capacity and skills through training and supportive supervision.

As part of the project, EngenderHealth also partnered with humanitarian organizations and other stakeholders working in the region, including the United Nations Population Fund (UNFPA), United Nations Office for the Coordination of Humanitarian Affairs, Family Guidance Association of Ethiopia, Mercy Corps, Marie Stopes Reproductive Choices, World Health Organization, and the World Food Programme. To align project activities with national activities, at the federal level EngenderHealth worked with the Ministry of Health, regional health bureaus, woreda (third-level administrative division) health offices, and Ethiopia’s different public health and emergency response structures.

Through these activities, we noted increases in most types of service utilization among project-supported facilities (Figure). Notably, data from project-support facilities showed that the number of clients provided with SRH services increased by 2.5-fold during the first year of the project, from a total of 700 SRH services provided in Quarter 1 (January 2020–March 2020) to 1,777 SRH services provided in Quarter 4 (October 2020–December 2020). Overall, project-supported facilities provided 5,441 women and girls (45% of young people aged 15–24 years) with SRH services, including antenatal care (N=2,657), skilled delivery (N=1,047), voluntary contraception (N=1,537), and clean home delivery (N=143). We observed just 1 case of GBV service uptake (not shown).

**FIGURE:**
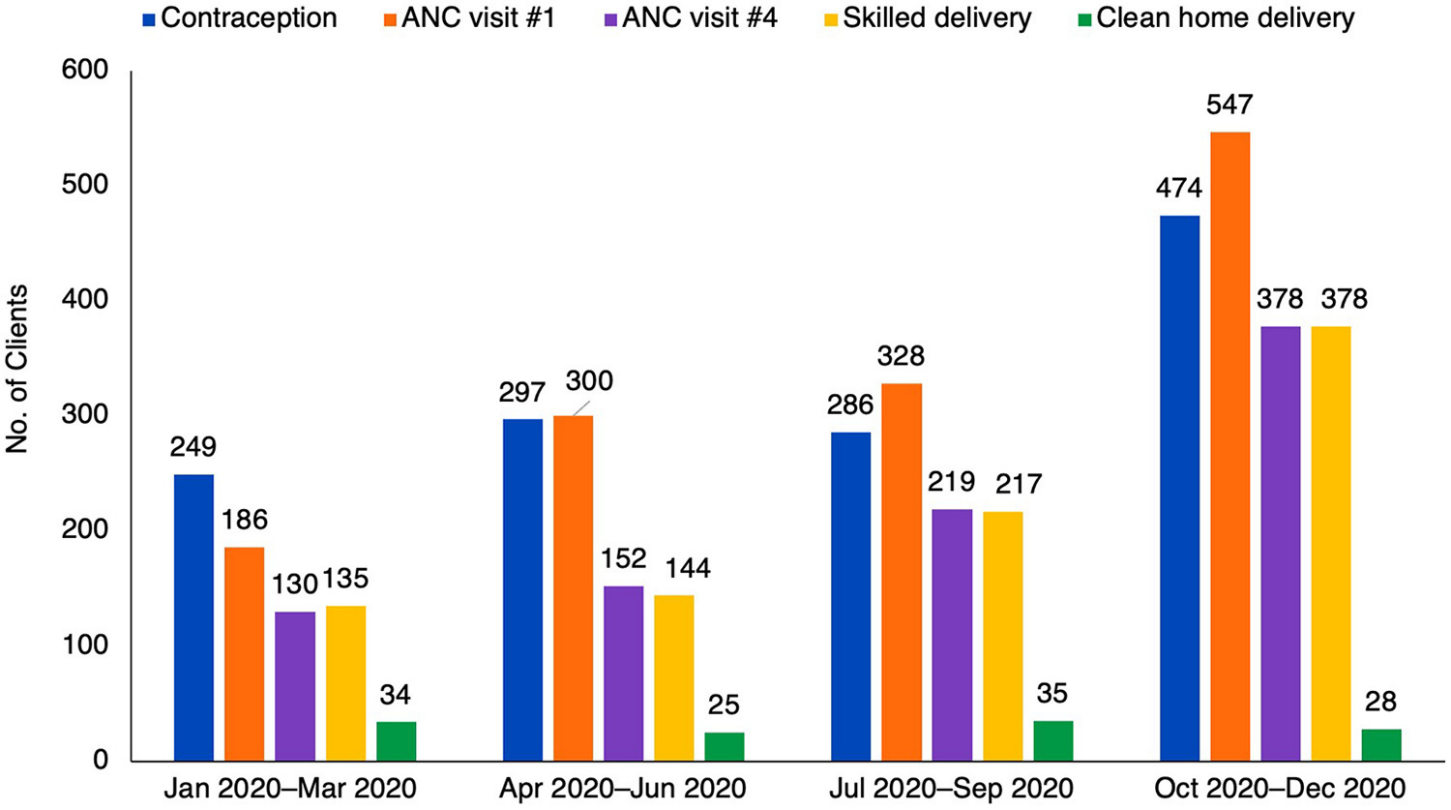
Monthly SRH Service Utilization by Type of Services at Project-Supported Health Facilities, Somali Region, Ethiopia, January 2020–December 2020^a^ Abbreviations: ANC, antenatal care; SRH, sexual and reproductive health. ^a^For the ease in interpreting trends, we present these increases as quarterly data from the 2020 calendar year, but in the text, we also reflect on monthly data collected in December 2019 and January 2021 to illustrate the project reach throughout the implementation period. Clean home delivery was conducted by trained traditional birth attendants whenever skilled delivery at an institution is not possible.

## METHODS

### Study Area

The independent study team (contracted by EngenderHealth) implemented the process evaluation in the Fafan Zone of the Somali region of Ethiopia. The IDP crisis is particularly pronounced in the Somali region of Ethiopia, where an estimated 896,000 climate-induced and conflict-affected IDPs resided across 419 displacement sites when the project was initiated.^19^ The largely rural Somali region spans over 350,000 square kilometers, is the second-largest region in the country, and presents complex resource and service delivery challenges.

The number of clients provided with SRH services increased by 2.5-fold during the first year of the project.

### Study Design and Period

The independent study team implemented the qualitative process evaluation from January 28, 2021 to February 26, 2021, using 4 focus group discussions (FGDs) and 13 key informant interviews (KIIs). The FGDs and KIIs assessed the progress and contributions of the project and documented lessons learned.

### Sample

To demonstrate study rigor, the team used a maximum variation sampling technique to include multiple perspectives of people involved in designing, implementing, and monitoring the SRHR project. The maximum variation sampling technique is the form of nested purposive sampling, where people that are involved in the different steps and activities of the project are purposively identified and invited for a qualitative inquiry. Accordingly, the study team included a range of participants for the FGDs or KIIs. These included community members that had been exposed to the project, community health volunteers (CHVs), community volunteers (elders, youth group leaders, and religious leaders), health care providers, camp coordinators, EngenderHealth staff, the project donor, and government partners. We implemented a total of 4 FGDs: 2 FGDs with female project beneficiaries (7 or 8 participants per group) and 2 FGDs with a mix of community volunteers (7 participants per group) (Table 1). We completed 13 KIIs with the following participants: 2 health care providers, each from different health care centers; 2 camp coordinators; 1 Somali Region’s Public Health Emergency Operations coordinator; 1 woreda health official; 1 Public Health Emergency Management (PHEM) director; 2 EngenderHealth project staff; 1 Somali regional office project staff; 1 Packard Foundation representative; 1 World Health Organization Ethiopia representative, and 1 UNFPA Ethiopia representative (Table 2). The mean age of the FGD participants was 30.6 years (community volunteers) and 27.8 years (project beneficiaries). Overall, 71% of the key informants were male.

**TABLE 1.** Characteristics of Focus Discussion Group Participants

**Focus Group**	**Participants**	**Mean Age, Years**	**Female, No. (%)**	**Total No. of Participants**
1	Project beneficiaries – group 1	31.4	8 (100)	8
2	Project beneficiaries – group 2	29.7	8 (100)	8
3	Community volunteers – group 1	27.4	3 (42.9)	7
4	Community volunteers – group 2	28.2	3 (42.9)	7

**TABLE 2.** Characteristics of Key Informant Interview Participants

**Participants**	**No.**	**Male, No. (%)**
Health care providers	2	2 (100)
Camp coordinators	2	2 (100)
Somali Region’s Public Health Emergency Operations coordinator	1	1 (100)
Woreda health official	1	1 (100)
National Public Health Emergency Management director	1	0 (0)
EngenderHealth project staff	2	1 (100)
Somali regional office project staff	1	1 (100)
Packard Foundation representative	1	0 (0)
World Health Organization Ethiopia representative	1	1 (100)
United Nations Population Fund Ethiopia representative	1	1 (100)

### Study Instruments

The independent consultant developed the FGD and KII guides, which were reviewed by the SRHR-IDP project team before administration. The FGD guides for project participants explored their perceptions of the availability and acceptability of the SRH services, and factors that affected community utilization of SRH services. Participants were also asked about the extent to which they benefited from the project, how successful or unsuccessful the project was in meeting their SRHR needs, and what should be done in the future. For CHV participants, the FGDs focused on their experiences with project implementation, including what went well, what did not go as planned, and the remaining challenges. The KII guides also focused on lessons learned during project implementation, reflections on the effective coordination of SRHR activities with humanitarian response and insights on the roles of different actors, and recommendations for future programming. The study team developed the instruments in English, translated these into Amharic, and pilot tested them before the study implementation.

### Data Collection and Procedures

The study team comprised a lead independent qualitative research and an experienced qualitative research assistant. The study team selected the participants purposively, using the maximum variation sampling technique. The method assisted with identifying key people with rich experiences and knowledge regarding the evaluation questions and ensured saturation of the themes being investigated.

To identify FGD participants, the study team used snowball sampling methods starting with community leaders in each district. The community leaders helped to identify women in the area who might have accessed SRH services or CHVs. The study team asked potential participants if they knew of other people who had accessed services or knew of other CHVs. The team identified participants in several ways: (1) EngenderHealth provided the list of project staff, nongovernmental organizations, and federal government office staff, (2) the Somali Regional Health Bureau provided the list of potential informants at the regional level, (3) the woreda health offices identified their staff involved in the project planning and implementation, and (4) IDP site coordinators identified relevant staff. Following this, the lead researcher and the qualitative research assistant reached out to the potential key informants and invited them to take part in the study. The study team did not provide any incentives for participation.

The study team implemented the FGDs and KIIs. The lead researcher trained the research assistant in the study procedures and data management. The team implemented all FGDs in person at a community center, while 11 KIIs were conducted using virtual platforms and 3 were conducted in person.

### Data Analysis

The lead researcher and qualitative research assistant transcribed the interviews verbatim and had the transcripts translated into English for analysis. The researchers relistened to the audio recordings to familiarize themselves with the data, confirm the contents were correctly transcribed, and verify that there were no major pieces of missing information in the transcripts. The lead researcher, who was bilingual, also read and reviewed each translated English transcript, comparing it against the original Amharic version. The study team analyzed the data using the framework methods for the analysis of qualitative data in multidisciplinary health research.^20^ The steps involved included transcription, familiarization with the interview, coding, developing, and applying the framework to assess low-, medium-, and high-level themes, and data interpretation. The coding and development of themes were assisted with NVivo 12 qualitative data analysis software. The lead researcher created a codebook (providing a framework for the analysis) and coded the data line-by-line in NVivo. The study team analyzed the frequency with which individuals reported themes, and to what extent the FGDs or KIIs mentioned these themes to identify patterns in the data. This procedure helped clarify which themes consistently emerged across all groups and which were idiosyncratic. The lead researcher aligned the themes that emerged from the data into the precoded themes identified from the FGD and KII guides (a priori research questions) and made constant comparisons until the final themes of the framework were achieved. EngenderHealth researchers read a selection of the transcripts and coding to validate the analysis and findings of the study. All researchers then presented on common themes that emerged through the analysis, rather than those findings that were mentioned by only 1 or 2 respondents.

### Ethical Approval

Ethical approval was obtained from the Somali Regional Health Bureau’s Research Ethics Committee to conduct the case study. The team interviewed participants in private spaces to maintain confidentiality and protect the safety of the study team and participants, while adhering to the National COVID-19 Prevention and Control Guidance. All data were anonymized and stored in password-protected files accessible by the lead researcher and EngenderHealth. EngenderHealth stored the data at the EngenderHealth office. The study team explained the research objectives to the participants before they gave their written consent. Key informants consented to information on their position and role being included in any relevant transcripts.

## RESULTS

### Partnership and Stakeholder Engagement

Respondents stressed that establishing strong partnerships and engaging relevant stakeholders from the inception to implementation and evaluation of the project assisted with creating an enabling environment for improving access to and utilization of SRH services among IDPs. Partner collaborations helped to avoid duplication of efforts, fill gaps in SRH services, procure the necessary supplies, and coordinate the needs of the target population. For instance, the health system staff reported that they were involved in planning and implementing project activities and monitoring and evaluation. Partnering with other non-governmental organizations enabled harmonization of activities, resource sharing, and joint performance evaluation, creating a platform to complement rather than compete. Respondents described how meaningful partnerships at the local and regional levels were central to enabling the project to adapt SRHR programs to the IDP setting. Similarly, respondents cited how these partnerships fostered alignment with other interventions for IDPs such as food, shelter, and education, and helped to avoid or reduce delays in carrying out project activities. For example, EngenderHealth’s collaboration with UNFPA assisted with project setup and procurement of emergency SRH kits. The project also signed a memorandum of understanding with the Somali region's Public Health Emergency Operations Center and worked with the regional Bureau of Disaster Preparedness and Risk Management to identify the IDPs' needs.

*We assisted them [SRHR-IDP project] during proposal development and provided the necessary materials. We continued our engagement during the implementation and monitoring. So, I can say we participated in the overall project implementation, from the beginning to the end.* —UNFPA Emergency Response Coordinator, Ethiopia

Strong partnerships and stakeholder engagement were key to supporting an enabling environment for SRH services for IDPs.

Respondents also described how the close involvement of regional- and local-level health staff in the implementation of project activities was central to establishing important relationships and ensuring smooth implementation, as well as supervision and monitoring activities.

*They [SRHR-IDP project] signed the project's MOU with us [Somali Regional Health Bureau] and implemented the project based on our agreement. They implemented activities aimed to build health facilities' capacity to increase institutional delivery and other SRH services. The project sent performance and progress reports to the RHB [Regional Health Bureau] and sometimes conducted joint supervision to the health facilities. Their main stakeholders were the RHB and woreda health offices, and I found their work relationships with us and the spirit of collaboration excellent.* —PHEM Center Coordinator, Somali Region

Another key theme to emerge was how the partnerships and networking fostered momentum for advocacy and the creation of an enabling environment for SRH services within the IDP humanitarian response. Respondents cited how participation in technical working groups and other fora was critical to developing and conveying a uniform agenda and advocacy messages to the Ethiopian Ministry of Health and the international community concerning the SRHR needs of IDPs. For instance, advocacy efforts resulted in including SRH for IDPs in the national plans.

Despite this, the KIIs illustrated that partnership efforts were also challenging. Respondents identified the need to improve the mapping of all essential stakeholders at project inception and to involve these stakeholders in all aspects of the project, from implementation to monitoring and evaluation. Respondents also cited that there needed to be greater involvement in the supervision and monitoring of the project by other stakeholders.

*I wish the project had engaged [Ethiopian Public Health Institute, EPHI] in the detail planning, monitoring, and evaluation of the activities. It would be good if they can support EPHI in implementing the SRH activities in IDP settings. They [SRHR-IDP project] need to improve collaboration with EPHI and other partners. It would be good to have a harmonized plan with budget lines shown. Also, good if they had an agreement signed with EPHI so that we can make strong follow up and perform activities jointly.* —PHEM Center Director, EPHI

### Inclusion of Local Government, Providers, and Community Members to Design and Develop Interventions

Our analysis suggests that the inclusion of local government actors, health providers, and community members across the design and implementation of project activities was important for delivering time-sensitive, context-specific SRH services to IDPs in crisis. Respondents emphasized the importance of addressing and refining the project objectives and activities with local health system actors and community stakeholders throughout different steps of the project. A key theme to emerge was the relevance of the consultative meetings between project staff and different stakeholders (Ethiopia Ministry of Health, woreda health offices, regional health bureaus, health providers, and community leaders) during project planning. Respondents cited how these meetings helped to clarify the immediate needs of the IDP camps. Through these consultative meetings, respondents elicited that the project was able to identify (1) key SRHR topic areas for providers and (2) staff in need of training and essential commodities for SRH service provision.

*When partners came to our woreda, they assessed maternal health services and found out that it was poor, and we needed support. Then, they held a discussion with us on how to improve maternal health services. Then, we agreed on building healthcare providers' capacity on SRH services … The involvement of EngenderHealth and other partners was crucial. —*Head, Tuli Guled woreda health office

Respondents emphasized the importance of addressing and refining the project objectives stages.

Respondents also confirmed that the involvement of local health offices during initial planning meetings helped to identify and address the pressing community needs. Respondents described how the community mobilization efforts—through the involvement of influential community members, religious leaders, youth leaders, and community health workers—were important to mobilize communities to uptake services.

*We closely work with the traditional birth attendants and community volunteers such as religious leaders, elders, and youth. Since they are trained on the SRH services, they are working as a channel that connects the community with us.* —Health care provider, Darima Health Center

### Prioritizing SRHR Programming and Implementation in IDP Settings

While acknowledging its challenges, respondents cited that prioritizing SRHR programming in IDP settings fills a key gap that is often overlooked in humanitarian response.

Respondents cited that prioritizing SRHR programming in IDP settings fills a key gap that is often overlooked in humanitarian response.

Respondents described how humanitarian organizations in the region focused on the delivery of essential services and discussed how SRHR had not been prioritized in the Somali region before the project. Respondents reflected on several activities that demonstrated how the SRHR-IDP project assisted with prioritizing SRH services for IDPs by strengthening provider capacity through clinical trainings, supportive supervision, and mentorship to improve the delivery and quality of SRH services and by training and mobilizing CHVs who helped to create awareness of SRH services. Respondents cited that the project's adaptation of the minimum initial service package was an important and fruitful activity that helped to contribute to the observed access to a wider range of contraceptives.

When asked about what respondents believed was most successful, many cited strengthening of service delivery and community mobilization efforts, believing that these approaches provided an important intervention model for SRH integration into IDP settings.

*The most unique contribution of the project partnerships and implementation activities was expanding access to and utilization of family planning services among the IDPs as well as the non-IDP community. Before this project we didn’t pay much attention to the family planning services be it awareness creation or improving access. However, the project connected us with the community in the SRH services. The other unique activity was the extensive community awareness creation activity conducted. Their effort assisted in dispelling community misconceptions and unfounded beliefs about family planning. —*Head, Tuli Guled woreda health office

Many respondents cited strengthening of service delivery and community mobilization efforts as the most successful approaches.

Despite efforts at the community level, FGD project beneficiaries described several persistent barriers to using SRH services, including misconceptions, stigma and discrimination, the need for spousal permission, religious beliefs, and fear of side effects and infertility.

*When community volunteers go out for community mobilization, they always face resistance from some community members who believed family planning was not acceptable in Islam. They said Allah decides the fate of everybody’s life. There was no need to use family planning methods.* —Community volunteer FGD participant, Qoloji IDP camp

*Among the issues that deter women in IDP from accessing reproductive health services is fear of contraceptives side effects despite lack of enough food in the household. There are also negative beliefs regarding birth spacing—some women believe that using pills for birth spacing could negatively affect their fertility in the future. Yes, these factors had a strong impact and affected women’s access to reproductive health services in the IDPs site, and we are working hard to dispel the misconceptions.* —Community volunteer FGD participant, Qoloji IDP camp

*There is 1 problem for most women in IDPs in accessing the SRH services. Women cannot decide to use SRH service by themselves without the consent of the husband. The women use the SRH services if the husband allows, and they don’t if he doesn’t.* —Female beneficiary FGD participant, Tuli Guled

Respondents also noted that GBV services were undoubtedly underutilized by community members. Even with community awareness–raising activities and training of health care workers on GBV survivor care, uptake of care by survivors at health facilities was very low. This low uptake may have been a result of negative community norms, though data on the reasons for this were limited.

*The GBV care services reporting, and utilization was not up to our expectations even though we trained the health workers and volunteers. There are not many people coming forward and reporting GBV experienced. This is also true in the [Demographic and Health Surveys] report. Only a few women report GBV to the health facilities. What we assume is that the community norms to access services are not favorable and is affecting uptake.* —Country representative, EngenderHealth, Ethiopia

An additional challenge of the project cited by the respondents was that activities were insufficient to meet the high levels of demand for SRH services within the IDP community. Respondents described how the overall needs of this community went far beyond the reach of the project and required expanded and sustained project implementation.

*The project supported the health system by providing commodities like birth spacing pills and some medical equipment to improve sexual and reproductive health services. I think these supports were good, but they were not adequate compared to the high needs.* —CHV FGD participant, Qoloji IDP camp

*In terms of adequacy, it can't be said it was sufficient because they worked in a few selected IDP sites, and there are many IDPs not covered with the project. But they worked according to their plan in their target kebeles.* —PHEM Center Coordinator, Somali Region

Respondents cited a challenge that activities were insufficient to meet the high levels of SRHR need within the IDP community.

This finding was compounded by other challenges related to provider trainings and clinical coaching. Despite efforts to collaborate with local officials to identify appropriate trainees during the project inception, respondents cited that the identified trainees did not have the expected minimum skills required for the training topics. Other challenges to emerge included an insufficient number of providers for training, high staff turnover, shortage of health care workers, and persistent gaps in infrastructure, equipment, and supplies. With limited budget and staff, the project addressed priority SRH issues in selected IDP sites.

*The project trained health care providers on SRHR. The main challenge we faced in building health workers' capacities was that, sometimes, the health facilities sent inappropriate health care providers for the SRHR pieces of training. … For instance, it is common to see that the health workers trained in the last 6 months have left the facility for better careers. So, this is a long-standing problem in this area.* —PHEM Center Coordinator, Somali Region

*I think they had an insufficient budget, and the number of the project staff was minimal, and the community promoters were employed only for a few months or so. The logistics shortages were other challenges we faced; sometimes, we observed delays in getting the SRH kits and other relevant supplies. —*PHEM Center Coordinator, Somali Region

Finally, the emergence of COVID-19 impacted the course of the SRHR-IDP project in several ways and presented a myriad of challenges. COVID-19 negatively impacted the initial implementation and performance of the project. For example, some in-person activities were canceled in response to the national government’s regulations. While the project adapted to virtual platforms for meetings and training instead of in-person activities, low internet coverage in the region limited the use of this approach as well as ongoing insecurity issues.

*There were restrictions of movement because of security problems. We lost time due to transportation problems, instability, and security problems. COVID-19 also affected the performance. For instance, [EngenderHealth] could not carry out training and meetings due to restrictions due to COVID-19. This affected EPHI’s as well as different partners. —* PHEM Center Director, EPHI

### Project Flexibility and Adaptability

Our analysis found that implementing and managing SRHR projects in humanitarian settings requires flexibility and adaptability. Respondents described how key elements such as advanced readiness and flexibility of organizational systems, strong procurement processes, and adherence to safety and security guidelines were critical to elements of project achievements for an IDP setting. For example, respondents described the importance of being able to adjust organizational standard operating procedures to accommodate the changes required for a humanitarian response. Respondents also described the necessity of decentralization of the decision-making process to rapidly tailor responses and respond to shifting needs. Through this decentralization, regional-level staff were able to make quick decisions that aided in timely project implementation without having to wait for headquarters approval. Finally, respondents cited that having in-country experience was also critical for ensuring appropriate supplies and overcoming logistical challenges.

*When you plan for logistics for humanitarian response, you don’t have a lot of time to process it. If the logistics are delayed, then the SRH needs of the IDPs could not be met. The challenge was most of the SRH products needed to be imported and required at least 3 months of processing. However, the project had a comparative advantage because most of its projects focused on SRH, and we had some supplies already in the country. —* SRHR-IDP Project Director

## DISCUSSION

The SRHR-IDP project sought to provide comprehensive SRHR knowledge and services to IDPs in the Somali region of Ethiopia. Our evaluation demonstrates how development organizations, such as EngenderHealth, can help respond to these emerging crises by making program adaptations and pivots in strategy in response to challenges to meet the complex needs of affected populations and learning from those challenges.

Our evaluation demonstrates how development organizations can help respond to emerging crises by making program adaptations and pivots in strategy to meet the complex needs of affected populations.

The following subsections summarize the main findings across several thematic areas (partnerships and collaboration, design of activities, alignment with national priorities) and conclude with a focus on the implementation challenges related to GBV services.

### Partnerships and Collaboration

Our findings illustrate how the provision of adequate SRH services for IDPs in humanitarian settings requires extensive partnerships and collaboration between development and humanitarian programs and local actors. Partnerships between development organizations, humanitarian response organizations, and the local government are critical, as they provide a foundation for project implementation, establishment of synergies and opportunities with other actors operating in the geographical areas, and assurance of local government support. Linked to this collaborative effort was the importance of establishing relationships with local government actors, health providers, and community members to help aid the design and implementation of project activities, so these were not only tailored to our target populations but met the needs of the project beneficiaries. These partnerships opportunity to meet the comprehensive health needs of IDPs, including SRHR needs, which are often not addressed in crisis settings in the same way as shelter; food; and water, sanitation, and hygiene services. This is consistent with other research that has highlighted the value of building strong, dynamic, flexible, risk-informed, and context-specific partnerships, and including local governments, to assist in navigating humanitarian spaces.^21^

Our findings are consistent with other evidence that shows that when humanitarian and development organizations collaborate, different approaches can be used to initiate timely, coordinated, and sustainable interventions that meet the diverse needs of people in crises.^22^ Through the SRHR-IDP project, we focused on the development and leveraging of partnerships to effectively prioritize and facilitate provision of SRH services. The findings highlighting the importance of organizational partnerships have been shared by other programs working in the combined humanitarian-development space.^23^^,^^24^ Our evaluation demonstrated that these partnerships are critical to address the limited technical capacity of humanitarian organizations to respond to SRHR needs. For example, engagement with humanitarian agencies and the local government helped strengthen their capacity to coordinate provision of a broader range of programming and services to meet the needs of IDPs. At the same time, these partnerships helped to build the project’s ability to adapt traditionally development-oriented approaches to work in humanitarian contexts through a coordinated response. While we were not able to assess the extent to which communities perceived the benefits and impact of these partnerships given the short evaluation period, we note that this is an area for further research.

### Design of Activities

An increasing evidence base recognizes the importance of cocreation in global health implementation research.^25^ As cited elsewhere, meaningful involvement of local stakeholders, including joint identification of needs and vulnerabilities, is essential for operationalizing coordinated, tailored, and effective responses.^26^ However, few studies have examined cocreation for implementation in humanitarian contexts, and those that have tend to be focused on end users alone and not broader stakeholders such as health systems staff and partners.^26^^,^^27^ Our findings contribute to this gap in evidence and emphasize the importance of cocreation of SRHR programs in humanitarian contexts. We observed that cocreation of projects and community involvement were crucial for strengthening resilient local capacity and ensuring risk-informed, context-specific implementation. The results of our evaluation illustrate how the use of a cocreation process, such as consultative meetings between project staff and different stakeholders (community leaders, health facilities, woreda health offices, regional health bureaus, and the Ethiopia Ministry of Health), helped to clarify the immediate needs of the IDP camps and was central to some of the project achievements.

In addition, the findings illustrate that community involvement and mobilization were critical to the project’s implementation, particularly in terms of SRHR information dissemination through CHVs and connecting girls and women to services through trusted community leaders. This finding aligns with recommendations from other studies in IDP settings that recognize the importance of community engagement and mobilization approaches for addressing SRHR needs.^28^ The training and mobilization of CHVs was a key activity that helped to reach communities with messages and awareness of SRH services. Consistent with a growing body of literature, our study illustrates persistent challenges in meeting the complex needs of IDPs.^29^^,^^30^ The project was not able to identify enough providers to ensure SRH services could be delivered throughout the duration of the project. Due to high staff turnover, fewer trained providers were still operating as the project progressed. In addition, the diffusion of messages in the community was questionable. Although CHVs were identified as an important means to reach communities, it is unclear whether the diffusion of their messages resulted in a shift in misconceptions or social norms. Indeed, our findings illustrated that there were still several persistent barriers related to the use of SRH services, including misconceptions, stigma, and discrimination. Our project was also challenged to reach survivors of GBV. The implementation of this activity and how best to reach survivors must be reevaluated as part of designing future interventions. These points are further discussed herein.

Training and mobilization of CHVs was a key activity that helped to reach communities with messages and awareness of SRH services.

### Engagement With National and Global Priorities

We also observed that effective SRHR response in prolonged and rapidly changing humanitarian situations requires strong engagement and linkages with national and global priorities, as has been reported by similar projects globally.^21^^,^^24^ Our evaluation highlighted the importance of ensuring that development organizations and their partnerships not only have in-country expertise but that their vision and activities are in alignment with national and global priorities. In this project example, our partnerships allowed us to advocate for the inclusion of SRH services in humanitarian response with the government, providing us with the foundation and scope to implement essential activities. We attribute this achievement to our partnerships and the persistent and early engagement with national and local governments—this helped us to adapt our approaches to meet humanitarian contexts, maximize our advocacy opportunities for the inclusion of other essential SRH services in response packages, and leverage our resources to best meet the current and future needs of IDPs and their host communities.

### Challenges Accessing GBV Services

This project attempted to improve access to GBV services but observed several challenges, as demonstrated by the low uptake of services. To further understand these challenges, the project implemented a qualitative follow-up study among IDP respondents in the Somali region that revealed several barriers: knowledge about GBV within the communities surveyed was limited and several types of GBV—including child early and forced marriage, domestic violence, and female genital mutilation and cutting—were considered normal cultural practices. The study also revealed that many GBV cases were unreported, often due to fear of stigma and repercussions, lack of trust in the justice system, and the preference for family- and community-level mediation. This is consistent with other research that illustrates low service uptake among GBV survivors may be attributed to barriers faced by survivors, such as fear of repercussions and cultural norms around domestic violence.^31^^,^^32^ These factors merit further exploration and are helpful building blocks for adapting future SRHR programs to reach GBV survivors.

### Limitations

This study presents findings specific to the IDP setting in Ethiopia. We acknowledge that there are several differences between an IDP setting, which is protracted but may experience rapid changes, and a new emergency setting or even a stable refugee setting.^1^ Recommendations such as partnerships and collaboration, design of interventions, and community activities may be more pertinent to an IDP setting, where the project was implemented, than to that of immediate crises. In short, while the results can inform SRHR program implementation for IDP populations in similar humanitarian settings, their external generalizability is limited. We also acknowledge that our FGD assessment was not designed to reach theoretical saturation and thus additional thematic areas may not have emerged. However, to address this, we used a maximum variation sampling technique for selecting study participants to reduce omission of the people with rich experiences of and involvement in the project activities. We also observed conflicting views that misconceptions were changed because of the community activities. This merits further research. Finally, the process evaluation was designed to assess the impact of the project-specific activities. However, the results should be interpreted in the context of the multiple implementing partners that made the successes and achievements a reality.

## CONCLUSION

The unmet need for comprehensive SRH services within the IDP population in Ethiopia is extremely high and prioritizing SRHR is critical. The SRH services provided through this project must be continued and integrated into the ongoing humanitarian response. While the project increased SRH service access and utilization, there are still large, unmet needs among IDPs in the Somali region. Our evaluation demonstrates several valuable lessons that contribute to the humanitarian-development nexus model and can be adapted by other development organizations working in a humanitarian setting. Engaging in meaningful partnerships with other organizations that are working with IDP populations will enable both development and humanitarian organizations to better provide for IDPs and their host communities—both during and beyond the immediate crisis—through collaborative and inclusive response. In addition, prioritizing community mobilization and engaging local stakeholders from project formulation through evaluation is critical, as well as training and mentoring health facility-level staff to ensure quality, lifesaving care for IDPs. These lessons identified by those closest to the project—including IDPs who received services, health facility staff, local government partners, and partner organizations working in this space—offer a path for effective prioritization of SRHR within humanitarian responses to internal displacement.

## Notes

**Peer Reviewed**

## References

[1] Internal Displacement Monitoring Centre (IDMC). *Global Report on Internal Displacement 2021*. IDMC; 2021. Accessed March 25, 2022. https://www.internal-displacement.org/global-report/grid2021/

[2] International Organization for Migration (IOM). *Ethiopia National Displacement Report 8: Site Assessment Round 25 & Village Assessment Survey Round 8: March–April 2021*. IOM; 2021. Accessed March 25, 2022. https://displacement.iom.int/reports/ethiopia-%E2%80%94-national-displacement-report-8-march-%E2%80%94-april-2021

[3] Ojengbede O Babawarun T Olayiwola O Ogun M Kongnyuy E Adorin O. Sexual and gender-based violence in camps for internally displaced people and host communities in northeast Nigeria: a mixed methods study. Lancet Glob Health. 2019;7:S6. 10.1016/S2214-109X(19)30091-9

[4] Muhumed AA Stites E Alexion E Burns D. *Livelihood Components of Durable Solutions for IDPs: Assessment of Three Cases in Somali Region, Ethiopia*. Tufts University, Feinstein International Center; 2021. Accessed September 28, 2022. https://reliefweb.int/report/ethiopia/livelihood-components-durable-solutions-idps-assessment-three-cases-somali-region

[5] Ethiopian Public Health Institute (EPHI), Ethiopia Federal Ministry of Health, ICF. *Ethiopia Mini Demographic and Health Survey 2019: Key Indicators*. EPHI/ICF; 2019. Accessed March 25, 2022. https://www.ephi.gov.et/images/Mini-Demographic-and-Health-Survey-Key-Indicators-2019.pdf

[6] Titiyos A Hailegebriel T Habte M. *Sexual and Reproductive Health Knowledge, Attitudes, and Practices among Internally Displaced Persons in the Somalia Region of Ethiopia: Baseline Assessment*. EngenderHealth; 2020. Accessed March 25, 2022. https://www.engenderhealth.org/wp-content/uploads/imported-files/Sexual-and-Reproductive-Health-Knowledge-Attitudes-and-Practices-among-Internally-Displaced-Persons-in-the-Somalia-Region-of-Ethiopia.pdf

[7] Amodu OC Richter MS Salami BO. A scoping review of the health of conflict-induced internally displaced women in Africa. Int J Environ Res Public Health. 2020;17(4):1280. 10.3390/ijerph17041280. 32079235 PMC7068277

[8] Barot S. In a state of crisis: meeting the sexual and reproductive health needs of women in humanitarian situations. Guttmacher Policy Rev. 2017;20:24–30. Accessed September 28, 2022. https://www.guttmacher.org/sites/default/files/article_files/gpr2002417_1.pdf

[9] United Nations (UN). *COVID-19 and People on the Move*. UN; 2020. Accessed September 28, 2022. https://ethiopia.un.org/en/48154-covid-19-and-people-move

[10] Amen Mohammed Ahmed W Boutros Shokai S Hassan Abduelkhair I Yahia Boshra A. Factors affecting utilization of family planning services in a post-conflict setting, South Sudan: a qualitative study. AIMS Public Health. 2015;2(4):655–666. 10.3934/publichealth.2015.4.655. 29546129 PMC5690433

[11] Singh NS Smith J Aryasinghe S Khosla R Say L Blanchet K. Evaluating the effectiveness of sexual and reproductive health services during humanitarian crises: a systematic review. PLoS One. 2018;13(7):e0199300. 10.1371/journal.pone.0199300. 29980147 PMC6035047

[12] Hakamies N Geissler PW Borchert M. Providing reproductive health care to internally displaced persons: barriers experienced by humanitarian agencies. Reprod Health Matters. 2008;16(31):33–43. 10.1016/S0968-8080(08)31349-4. 18513605

[13] Howe P. The triple nexus: a potential approach to supporting the achievement of the Sustainable Development Goals? World Dev. 2019;124:104629. 10.1016/j.worlddev.2019.104629

[14] Konyndyk J. *Fit for the Future: Envisioning New Approaches to Humanitarian Response*. Center for Global Development; 2018. Accessed March 25, 2022. https://www.cgdev.org/sites/default/files/Konyndyk-Fit-for-the-Future.pdf

[15] Mendenhall M. *Navigating the Humanitarian-Development Nexus in Forced Displacement Contexts*. UNICEF; 2019. Accessed March 25, 2022. https://www.unicef.org/esa/media/4866/file

[16] Nelson PJ Dorsey E. At the nexus of human rights and development: new methods and strategies of global NGOs. World Dev. 2003;31(12):2013–2026. 10.1016/j.worlddev.2003.06.009

[17] Barrow F. *Bridging the Reproductive Health Gap Between the Humanitarian and Development Nexus*. Master’s thesis. University of North Carolina at Chapel Hill; 2019. 10.17615/r3k4-nz09

[18] Bronfenbrenner U. Lewinian space and ecological substance. J Soc Issues. 1977;33(4):199–212. 10.1111/j.1540-4560.1977.tb02533.x

[19] UN-Habitat partner with sister agencies to support durable solutions for IDPs in Somali Region, Ethiopia. OCHA/ReliefWeb. February 25, 2021. Accessed March 25, 2022. https://reliefweb.int/report/ethiopia/un-habitat-partner-sister-agencies-support-durable-solutions-idps-somali-region

[20] Gale NK Heath G Cameron E Rashid S Redwood S. Using the framework method for the analysis of qualitative data in multi-disciplinary health research. BMC Med Res Methodol. 2013;13(1):117. 10.1186/1471-2288-13-117. 24047204 PMC3848812

[21] Food and Agriculture Organization of the United Nations (FAO), Development Initiatives (DI), the Norwegian Refugee Council (NRC). *Development Actors at the Nexus: Lessons From Crises in Bangladesh, Cameroon and Somalia, Synthesis Report*. FAO/DI/NRC; 2021. Accessed March 25, 2022. http://devinit.org/media/documents/Development_actors_at_the_nexus_Lessons_from_crises_in_Bangladesh_Cameroon_and_Somalia.pdf

[22] Fanning E Fullwood-Thomas J. *The Humanitarian-Development Peace Nexus: What Does It Mean for Multi-mandated Organizations*? Oxfam; 2019. Accessed March 25, 2022. https://reliefweb.int/sites/reliefweb.int/files/resources/dp-humanitarian-development-peace-nexus-260619-en_0.pdf

[23] Global Nutrition Cluster. *Lessons Learned from Humanitarian Development Nexus Reviews in Myanmar, Niger, and Afghanistan*. Global Nutrition Cluster; 2021. Accessed March 25, 2022. https://www.nutritioncluster.net/sites/nutritioncluster.com/files/2020-12/GNC%20SUN%20HDN%20Policy%20brief%20FINAL.pdf

[24] The Alliance for Child Protection and Humanitarian Action (Alliance). *“Humanitarian Development Nexus” and Child Protection: Sharing Responsibility for Children’s Protection – Addressing Risks and Vulnerabilities Through Cohesive Partnerships*. Alliance; 2019. Accessed March 25, 2022. https://www.socialserviceworkforce.org/system/files/resource/files/Background-Paper-Humanitarian-Development-Nexus.pdf

[25] Beran D Lazo-Porras M Cardenas MK. Moving from formative research to co-creation of interventions: insights from a community health system project in Mozambique, Nepal and Peru. BMJ Glob Health. 2018;3(6):e001183. 10.1136/bmjgh-2018-001183. 30498592 PMC6254743

[26] James LE Welton-Mitchell C Michael S. Development and testing of a community-based intervention to address intimate partner violence among Rohingya and Syrian refugees: a social norms-based mental health-integrated approach. Int J Environ Res Public Health. 2021;18(21):11674. 10.3390/ijerph182111674. 34770188 PMC8582911

[27] Bartlett R Boyle JA Simons Smith J Khan N Robinson T Ramaswamy R. Evaluating human-centred design for public health: a case study on developing a healthcare app with refugee communities. Res Involv Engagem. 2021;7(1):32. 10.1186/s40900-021-00273-2. 34053451 PMC8166144

[28] Improving sexual and reproductive health services among refugees and internally displaced people. Health Cluster/World Health Organization. October 7, 2020. Accessed September 28, 2022. https://healthcluster.who.int/newsroom/news/item/07-10-2020-improving-sexual-and-reproductive-health-services-among-refugees-and-internally-displaced-people

[29] Cantor D Swartz J Roberts B. Understanding the health needs of internally displaced persons: a scoping review. J Migr Health. 2021;4:100071. 10.1016/j.jmh.2021.100071. 34820657 PMC8600058

[30] Brookings Institution. *Improving the Protection of Internally Displaced Women: Assessment of Progress and Challenges*. Brookings Institution; 2014. Accessed March 25, 2022. https://www.brookings.edu/wp-content/uploads/2016/06/Improving-the-Protection-of-Internally-Displacement-Women-October-10-2014.pdf

[31] Odwe G Undie CC Obare F. Attitudes towards help-seeking for sexual and gender-based violence in humanitarian settings: the case of Rwamwanja refugee settlement scheme in Uganda. BMC Int Health Hum Rights. 2018;18(1):15. 10.1186/s12914-018-0154-6. 29530031 PMC5848542

[32] Muluneh MD Alemu YW Meazaw MW. Geographic variation and determinants of help seeking behaviour among married women subjected to intimate partner violence: evidence from national population survey. Int J Equity Health. 2021;20(1):13. 10.1186/s12939-020-01355-5. 33407515 PMC7789001

